# Cost-effectiveness of prostate cancer screening: a systematic review of decision-analytical models

**DOI:** 10.1186/s12885-017-3974-1

**Published:** 2018-01-18

**Authors:** Sabina Sanghera, Joanna Coast, Richard M. Martin, Jenny L. Donovan, Syed Mohiuddin

**Affiliations:** 1 Health Economics at Bristol, Population Health Sciences, Bristol Medical School, University of Bristol, Bristol, BS8 2PS UK; 2 Collaboration for Leadership in Applied Health Research and Care West at University Hospitals Bristol, Bristol, BS1 2NT UK; 3 Population Health Sciences, Bristol Medical School, University of Bristol, Bristol, BS8 2PS UK; 4 MRC Integrative Epidemiology Unit, University of Bristol, Bristol, BS8 2BN UK

**Keywords:** Cost-effectiveness, Prostate cancer, PSA test, Screening, Systematic review

## Abstract

**Background:**

There is ongoing debate about the harms and benefits of a national prostate cancer screening programme. Several model-based cost-effectiveness analyses have been developed to determine whether the benefits of prostate cancer screening outweigh the costs and harms caused by over-detection and over-treatment, and the different approaches may impact results.

**Methods:**

To identify models of prostate cancer used to assess the cost-effectiveness of prostate cancer screening strategies, a systematic review of articles published since 2006 was conducted using the NHS Economic Evaluation Database, Medline, EMBASE and HTA databases. The NICE website, UK National Screening website, reference lists from relevant studies were also searched and experts contacted. Key model features, inputs, and cost-effectiveness recommendations were extracted.

**Results:**

Ten studies were included. Four of the studies identified some screening strategies to be potentially cost-effective at a PSA threshold of 3.0 ng/ml, including single screen at 55 years, annual or two yearly screens starting at 55 years old, and delayed radical treatment. Prostate cancer screening was modelled using both individual and cohort level models. Model pathways to reflect cancer progression varied widely, Gleason grade was not always considered and clinical verification was rarely outlined. Where quality of life was considered, the methods used did not follow recommended practice and key issues of overdiagnosis and overtreatment were not addressed by all studies.

**Conclusion:**

The cost-effectiveness of prostate cancer screening is unclear. There was no consensus on the optimal model type or approach to model prostate cancer progression. Due to limited data availability, individual patient-level modelling is unlikely to increase the accuracy of cost-effectiveness results compared with cohort-level modelling, but is more suitable when assessing adaptive screening strategies. Modelling prostate cancer is challenging and the justification for the data used and the approach to modelling natural disease progression was lacking. Country-specific data are required and recommended methods used to incorporate quality of life. Influence of data inputs on cost-effectiveness results need to be comprehensively assessed and the model structure and assumptions verified by clinical experts.

**Electronic supplementary material:**

The online version of this article (10.1186/s12885-017-3974-1) contains supplementary material, which is available to authorized users.

## Background

Prostate cancer is the most common cancer in men in Europe and the second most common cancer in men worldwide. In 2012, 417,000 cases were diagnosed in Europe and 1,111,000 cases worldwide [1], so the disease has an important impact on healthcare resources. Symptomatic cases usually occur when the disease has metastasised and curative treatments are unlikely to be effective. A screen test, prostate-specific antigen (PSA) blood test, followed by a biopsy can be used to detect prostate cancers when asymptomatic and localised within the prostate gland, but PSA is not a specific marker for prostate cancer and prostate biopsy is associated with adverse effects [2]. Current diagnostic methods lead to over-detection of cancers that may not progress to become clinically important in a man’s lifetime, but can also miss aggressive, potentially fatal prostate cancers [3]. Treatments also have consequences. While the UK ProtecT trial of treatments for PSA-detected localised prostate cancer observed only 1% mortality in men with prostate cancer after a median 10-year follow-up, there were increased risks of disease progression and metastases following active monitoring [4] and impacts on urinary, sexual and bowel function from radical surgery or radiotherapy [5].

Large trials (ERSPC and PLCO) have quantified potential benefits from various screening strategies, but also confirmed considerable harms from overdiagnosis and overtreatment [6–8]. The US Preventive Services Task Force review, considering the totality of evidence, found limited prostate cancer-specific mortality benefit insufficient to outweigh the risks of overtreatment and harms [9]. However, to account for evidence from recent trials the recommendation is being revised to indicate a ‘potential’ benefit of reducing cancer-specific mortality. This potential benefit of screening may be observed in men whose prostate cancer is destined to progress. Other men are, on the other hand, subjected to unnecessary tests and treatments, which are costly both economically on healthcare resources and in harm caused to patients.

Policy decisions about whether the potential benefits of screening outweigh the costs and harms require a formal comparison of the costs and consequences of alternative courses of action through an economic evaluation. Many international institutes recommend cost-utility analysis, measuring health-related outcomes using quality-adjusted life-years (QALYs) combining length with quality of life measured by generic instruments (e.g. EQ-5D or SF-6D) to enable comparisons to be drawn across services [10–16]. As much of the benefit or harm arises from subsequent treatment, the value of any screening test is best understood by assessing the care pathway over a patient’s lifetime using decision analytic modelling [17].

Since the mid-1990s, several model-based cost-effectiveness analyses have been published for prostate cancer screening using different methods that may impact on results. The aim of this study was to systematically review model-based cost-effectiveness analyses to; (1) provide an overview of cost-effectiveness recommendations, (2) identify similarities and deviations in the evidence base and methods used, and (3) identify key issues to inform future analyses.

## Methods

### Search strategy

In April 2016, studies were identified by searching the NHS Economic Evaluation Database (EED), Medline, EMBASE, HTA databases, NICE guidelines, UK National Screening Committee guidance, reference lists from relevant studies and contacting experts. Search terms included free text and MESH terms (See Additional file 1 for search strategies).

The search was limited to English language publications and studies published between January 2006 and April 2016. An update was performed from April 2016–February 2017. Reports from NICE and UK National Screening Committee were considered as they are important inputs to UK decision-making and can inform practice in other countries. The review was restricted to evidence from January 2006 onward to reflect current practice in screening for prostate cancer and economic evaluation modelling methods.

The guidelines from the Centre for Reviews and Dissemination, PRISMA and Cochrane collaboration for reviews were followed [18–20].

### Inclusion criteria

Included studies reported: i) a model-based economic evaluation of any PSA screening strategy; or ii) natural history models of prostate cancer that were used to inform the model structure for cost-effectiveness analysis.

Studies evaluating any PSA strategy were considered. Men of any age and in any country were included. Any economic evaluation type was included.

### Study selection and data extraction

Study selection was performed independently by two reviewers (SS and SM): the first stage considered the relevance of the title and abstract, and the second involved reading the full text of potentially relevant papers. Relevant studies were carried forward for data extraction. 10% of the title and abstracts and all full text papers were reviewed by a second reviewer.

As the purpose of the review was to report the methodological approaches used in model-based economic evaluations, a formal quality checklist was not used to select studies, but relevant sections of an existing economic evaluation checklist along with recommendations from NICE were used to extract and evaluate studies [10, 21]. Key clinical issues, such as reporting of overdiagnosis were included (see Additional file 1 for data extraction criteria). Due to the nature of the review, a narrative synthesis of data was undertaken.

## Results

**Fig. 1:**
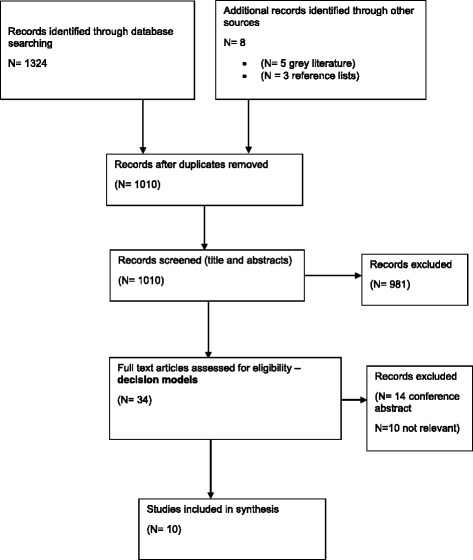
Flow diagram of study selection process

In total, 1324 studies were identified. After removing duplicates and checking for eligibility, 34 full text papers were retrieved (Fig. 1). Ten studies were included in the review: nine model-based economic evaluations were identified and one study identified the number needed to treat to identify a cost-effectiveness threshold [22]. The latter study was included as the model type, structure and parameters could potentially answer the review question.

### Study type

The study characteristics are presented in Table 1. Prostate cancer screening strategies were compared in several countries, including UK (*n* = 3), Australia (*n* = 2), US (*n* = 1), Canada (*n* = 1) and Europe (*n* = 1) with two studies not reporting the country setting.

**Table 1:** Study characteristics

Study	Setting	Population	Strategies compared	PSA threshold	Treatment	Outcome measure
Chilcott et al. [23]	UK	Men aged 50–74	· single screen at 50 · screen every 4 years from 50 to 74 years · screen every 2 years 50–74 years · screen every year from 50 to 74 · screen at 50, 60, 65, 70 · screen every 4 years 50–70, 55–74, 55–70 · screen every 2 years 50–70, 55–74, 55–70	3.0 ng/ml 3.0 ng/ml 3.0 ng/ml 3.0 ng/ml 3.0 ng/ml 3.0 ng/ml 3.0 ng/ml	· prostatectomy with ADT · radiotherapy with ADT · prostatectomy without ADT · radiotherapy without ADT · watchful waiting · active monitoring	Cost per QALY gained
Heijnsdijk et al. [25]	NR	Men aged 55–75	68 scenarios:	3.0 ng/ml	· radiotherapy	Cost per QALY gained
			· starting at age 55; screen intervals at 1, 2, 3, 4, 6, 8, 10, 12, 14 years	3.0 ng/ml	· prostatectomy	
			· once in a lifetime	3.0 ng/ml	· active surveillance	
			· age at stopping was varied 55–75 years	3.0 ng/ml	· metastases: palliative care	
Hummel and Chilcott [24]	UK	Men aged 50–74	· single screen at 50:	3.0 ng/ml	· radiotherapy (with and without hormone therapy)	Cost per QALY gained
			· screen every 4 years from 50 to 74 years	3.0 ng/ml	· prostatectomy (with and without hormone therapy)	
			· screen every 2 years 50–74 years	3.0 ng/ml	· watchful waiting	
			· screen every year from 50 to 74 years	3.0 ng/ml	· active monitoring	
Keller et al. [29]	Australia	Men aged 50–69	· opportunistic screening (current practice) · screen every 2 years from 50 to 69 years	3.0 ng/ml to 2.5 ng/ml^a^	· prostatectomy · radiotherapy · active surveillance	Cost per QALY gained
				3.0 ng/ml to 2.5 ng/ml^a^	· watchful waiting One strategy with immediate treatment and one delayed based on Gleason score	Cost per life year gained
Kobayashi et al. [27]	NR	Men aged 50–70	· annual screen irrespective of baseline	N/A	no details on exact nature of treatments	Cost per QALY gained
			· baseline PSA ≤ 1.0 ng/ml biennial rescreening	1.0 ng/ml		
			· baseline PSA ≤ 2.0 ng/ml biennial rescreening	2.0 ng/ml		
			· baseline PSA ≤ 3.0 ng/ml biennial rescreening	3.0 ng/ml		
			· baseline PSA ≤ 4.0 ng/ml biennial rescreening	4.0 ng/ml		
Martin et al. [30]	Australia	Men aged 50- (unclear)	· average risk: screen every 4 years	4.0 ng/ml	· radiotherapy (with and without hormone therapy)	Cost per QALY gained
			· high risk: screen every 4 years	4.0 ng/ml	· prostatectomy (with and without hormone therapy)	
			· very high risk: screen every 4 years	4.0 ng/ml	· conservative management	
Pataky et al. [26]	Canada	Men aged 40–74	14 scenarios:		· radiotherapy (with and without hormone therapy)	Cost per QALY gained and
			· Screen at 50, 60, 70	3.0 ng/ml	· prostatectomy (with and without hormone therapy)	
			· Screen at 60 followed by screen at 65	3.0 ng/ml	· conservative management	Cost per life year gained
			· screen every 4 years 55–69, 50–74	3.0 ng/ml		
			· screen every 4 years 50–74	3.0 ng/ml, (4.0 ng/ml for ≥70 years old)		
			· screen every 2 years 60–74, 50–69, 55–74, 50–74, 40–74	3.0 ng/ml		
			· screen every 2 years 50–74	3.0 ng/ml, (4.0 ng/ml for ≥70 years old)		
			· adaptive screen 50–74	3.0 ng/ml		
Roth et al. [28]	US	Men aged 45–69	18 scenarios:		(1) all cases receive curative surgery, radiotherapy with or without adjuvant hormone therapy,	Cost per QALY gained and
			· screen yearly 45–69, 50–74, 55–69	4.0 ng/ml	(2) Gleason < 7, <T2a receive conservative treatment or curative treatment, all others as above	
			· screen yearly 45–69, 50–74, 55–69	10.0 ng/ml		
			· screen yearly if >3.0 ng/ml, every 2 years otherwise,45–69	3.0 ng/ml		Cost per life year gained
			· screen yearly if >3.0 ng/ml, every 2 years otherwise,45–69	10.0 ng/ml		
			· screen every 4 years 50–74	4.0 ng/ml		
			· screen every 4 years 50–74, 55–69	10.0 ng/ml		
			· screen every 2 years if >1.0 ng/ml, every 4 years otherwise, 50–74	4.0 ng/ml		
			· screen every 2 years if >1.0 ng/ml, every 4 years otherwise, 50–74	10.0 ng/ml		
			· screen yearly with age dependent threshold, 50–74	3.5(50–59), 4.5(60–69), 6.5(70–74)		
			· screen yearly with age dependent threshold 50–74	4.5(50–59), 5.5(60–69), 8.5(70–74)		
			· screen every 2 years 55–69	3.0 ng/ml		
			· screen every 4 years 55–69	3.0 ng/ml		
			· screen every 2 years 55–69	10.0 ng/ml		
Wolstenholme et al. [31]	UK	Men aged 50–65	· single screen at 50, 55, 60 and 65	3.0 ng/ml	active surveillance, prostatectomy, radiotherapy, orchidectomy, hormonal therapy, chemotherapy	Cost per life year saved/gained
			· screen every 5 years 50–65	3.0 ng/ml		
Shteynshlyuger & Andriole, [22]	Europe	Men aged 60–70	· “PSA screening reported in ERSPC”:		· Not reported. Assumed follows ERSPC trial	Cost per life year saved
			· screen every 4 years	3.0 ng/ml or 4.0 ng/ml (depending on the centre)		
			· no such screening	3.0 ng/ml or 4.0 ng/ml (depending on the centre)		

^a^ The study is based on the Goteburg centre of the ERSPC trial, which altered the PSA threshold overtime to reflect current recommendations. *ADT* androgen deprivation therapy

Most of the studies reported a cost-utility analysis, where outcomes are presented in QALYs gained [23–30]. Three of these studies also reported outcomes in terms of life years gained or saved [26, 28, 29]. The remaining studies considered only reporting life years gained or saved [22, 31], but recommendations are difficult to interpret as a conventional threshold for determining cost-effectiveness has not been established based on cost per life-year gained [22, 31].

All included studies assessed the cost-effectiveness of a screening programme by considering the screen, test and treatment pathway for men over a lifetime (ranging from up to 80–100 years old), except one [29], which modelled up until 70 years old due to data availability.

### Screening strategies

All studies estimated the cost-effectiveness of more than one PSA screening strategy (Table 2). Screening interventions can be categorised accordingly: (1) Single screen (*n* = 5), (2) Repeat screens (*n* = 10), and (3) Adaptive screens (*n* = 3).

1. A single screen was assessed in all three UK studies at ages 50 [23, 24, 31], 55 [31], 60 [31], and 65 [31] years old. The Canadian study also assessed a single screen at ages 50, 60, and 70 years old [26], and Heijnsdijk et al. [25] assessed screening at ages 55–75 years old. All strategies used a PSA threshold of 3.0 ng/ml.
  Overdiagnosis rates, expressed as the percentage of prostate cancer diagnoses relative to prostate cancer deaths, for a single screen ranged from 0.06% (50 years), 1.9% (60 years), and 7.1% (70 years) [26]. Rates of 18% (50 years) were also reported, expressed as the proportion of men who died from other causes [23], and 29.7% (55 years) [25], expressed as the proportional change in cancer detected between the screen and no screen arms.
  When accounting for quality of life, a single screen was not shown to be cost-effective in three studies [23, 24, 26], but one study found that a single screen at 55 years old may be potentially cost-effective. ($31,470/ QALY gained) [25].
2. All studies considered repeat screens, including annual (*n* = 5), two (*n* = 6), four (*n* = 7), and five (*n* = 1) yearly intervals in a range of age cohorts from 40 to 75 years old (see Table 1 for all strategies). The starting age varied from 40 to 60 years old and the stopping age varied from 55 to 75 years old. Martin et al. [30] was the only study to compare 4 yearly screening results by risk and to assess only a PSA threshold of 4.0 ng/ml. The most common PSA threshold used was 3.0 ng/ml (n = 7), with one study comparing different thresholds for all men (3.0 ng/ml, 4.0 ng/ml, 10 ng/ml) [28].
  Overdiagnosis for repeat screens ranged from 8.4–21.9% [26], over 44% [23] and between 31.1%–36.7% [25].
  When accounting for quality of life, repeat screening was not shown to be cost-effective in four studies [23, 24, 26, 29]. However, two studies found some strategies to be potentially cost-effective: Screening every 4 years at ages 55–69 years old at a PSA threshold of 10 ng/ml ($92,450) [28], screening very high-risk men every 4 years at a PSA threshold of 4.0 ng/ml (AUS$30,570) [30], and yearly or two-yearly screens starting at 55 years old and stopping at ages ranging between 59 and 63 years old (see Table 2 for details) with incremental cost-effectiveness ratios (ICERs) ranging from $38,560–$76,910/QALY gained [25].
3. Three studies [26–28] assessed the cost-effectiveness of adaptive screen frequencies, where the subsequent screen interval was based on the baseline PSA level [26, 27] or the PSA threshold for biopsy was dependent on age [26, 28]. None of the strategies compared were shown to be cost-effective.
  Pataky et al. [26] reported overdiagnosis rates of 5.1% for a strategy where all men are tested at 60 years old, with men above the median screened again at 65 years old, and 21% for a strategy where men with a PSA above the age median are screened again in 2 years and others screened again in 4 years.

**Table 2:** Cost-effectiveness results for studies reporting QALYs

Study	Setting	Strategies compared	PSA threshold	ICER (Cost/QALY gained)	Threshold
Chilcott et al. [23]	UK	· single screen at 50	3.0 ng/ml	Dominated^a^	£20–30,000/QALY gained
		· screen every 4 years from 50 to 74 years	3.0 ng/ml	Dominated	
		· screen every 2 years 50–74 years	3.0 ng/ml	Dominated	
		· screen every year from 50 to 74	3.0 ng/ml	Dominated	
		· screen at 50, 60, 65, 70	3.0 ng/ml	Dominated	
		· screen every 4 years 50–70, 55–74, 55–70	3.0 ng/ml	Dominated	
		· screen every 2 years 50–70, 55–74, 55–70	3.0 ng/ml	Dominated	
Heijnsdijk et al. [25] Costs in US dollars	NR	68 scenarios (efficient strategies only):	3.0 ng/ml		No formal threshold
		*· single screen at 55 years*	*3.0 ng/ml*	*$31,467*	
		· screen at 55 and then 57 years	3.0 ng/ml	$38,563	
		· screen at 55 and then 58 years	3.0 ng/ml	$40,785	
		· screen every 2 years 55–59 years	3.0 ng/ml	$45,615	
		· screen every 2 years 55–61 years	3.0 ng/ml	$54,349	
		· screen yearly 55–61 years	3.0 ng/ml	$63,263	
		· screen yearly 55–62 years	3.0 ng/ml	$69,481	
		· screen yearly 55–63 years	3.0 ng/ml	$76,910	
Hummel and Chilcott [24]	UK	· single screen at 50	3.0 ng/ml	Dominated	£20–30,000/QALY gained
		· screen every 4 years from 50 to 74 years	3.0 ng/ml	Dominated	
		· screen every 2 years 50–74 years	3.0 ng/ml	Dominated	
		· screen every year from 50 to 74 years	3.0 ng/ml	Dominated	
Keller et al. [29]	Australia	· opportunistic screening (current practice)	3.0 ng/ml to 2.5 ng/ml		A$50,000/QALY gained
		· screen every 2 years from 50 to 69 years (immediate treatment)	3.0 ng/ml to 2.5 ng/ml	A$147,528	
		*· screen every 2 years from 50 to 69 years (AS for low risk cancer)*		*A$45,882*	
Kobayashi et al. [27] Costs in US dollars	NR	· annual screen irrespective of baseline, 50–70	N/A	$165,938	No formal threshold
		· baseline PSA ≤ 1.0 ng/ml biennial rescreening, 50–70	1.0 ng/ml	$46,505	
		· baseline PSA ≤ 2.0 ng/ml biennial rescreening, 50–70	2.0 ng/ml	$5925	
		· baseline PSA ≤ 3.0 ng/ml biennial rescreening, 50–70	3.0 ng/ml		
		· baseline PSA ≤ 4.0 ng/ml biennial rescreening. 50–70	4.0 ng/ml	Dominated	
Martin et al. [30]	Australia	· average risk screen: every 4 years, 50+	4.0 ng/ml	A$291,817	A$50,000/QALY gained
		· high risk screen: every 4 years, 50+	4.0 ng/ml	A$110,726	
		*· very high risk screen: every 4 years, 50+*	*4.0 ng/ml*	*A$30,572*	
Pataky et al. [26]	Canada	14 scenarios:			CAN $50–80,000/QALY gained
		· screen at 50, 60, 70	3.0 ng/ml	Dominated	
		· screen at 60 followed by screen at 65	3.0 ng/ml	Dominated	
		· screen every 4 years 55–69, 50–74	3.0 ng/ml	Dominated	
		· screen every 4 years 50–74	3.0 ng/ml, (4.0 ng/ml for ≥70 years old)	Dominated	
		· screen every 2 years 60–74, 50–69, 55–74, 50–74, 40–74	3.0 ng/ml	Dominated	
		· screen every 2 years 50–74	3.0 ng/ml, (4.0 ng/ml for ≥70 years old)	Dominated	
		· adaptive screen 50–74	3.0 ng/ml	Dominated	
Roth et al. [28]	US	18 scenarios: Contemporary treatment scenario			
		· screen yearly 45–69, 50–74, 55–69	4.0 ng/ml	Dominated	US$ 50,000-150,000/QALY gained typically referred to (study refers to $150,000/QALY gained)
		· screen yearly 45–69	10.0 ng/ml	US $326,292	
		· screen yearly 50–74	10.0 ng/ml	US $330,065	
		· screen yearly 55–69	10.0 ng/ml	US $300,884	
		· screen yearly if >3.0 ng/ml, every 2 years otherwise,45–69	3.0 ng/ml	Dominated	
		· screen yearly if >3.0 ng/ml, every 2 years otherwise,45–69	10.0 ng/ml	US $184,074	
		· screen every 4 years 50–74	4.0 ng/ml	Dominated	
		· screen every 4 years 50–74	10.0 ng/ml	US $170,195	
		*· screen every 4 years 55–69*	*10.0 ng/ml*	*US$92,446*	
		· screen every 2 years if >1.0 ng/ml, every 4 years otherwise, 50–74	4.0 ng/ml	Dominated	
		· screen every 2 years if >1.0 ng/ml, every 4 years otherwise, 50–74	10.0 ng/ml	US $209,338	
		· screen yearly with age dependent threshold, 50–74	3.5(50–59), 4.5(60–69), 6.5(70–74)	Dominated	
		· screen yearly with age dependent threshold 50–74	4.5(50–59), 5.5(60–69), 8.5(70–74)	Dominated	
		· screen every 2 years 55–69	3.0 ng/ml	Dominated	
		· screen every 4 years 55–69	3.0 ng/ml	Dominated	
		· screen every 2 years 55–69	10.0 ng/ml	US $170,981	
		Selective treatment scenarios			
		· screen yearly 45–69	4.0 ng/ml	US $163,214	
		· screen yearly 50–74	4.0 ng/ml	US $243,768	
		· screen yearly 55–69	4.0 ng/ml	*US $128,680*	
		· screen yearly if >3.0 ng/ml, every 2 years otherwise,45–69	3.0 ng/ml	US $313,214	
		· screen every 4 years 50–74	4.0 ng/ml	*US $89,333*	
		· screen every 2 years if >1.0 ng/ml, every 4 years otherwise, 50–74	4.0 ng/ml	*US $136,332*	
		· screen yearly with age dependent threshold, 50–74	3.5(50–59), 4.5(60–69), 6.5(70–74)	US $166,784	
		· screen yearly with age dependent threshold 50–74	4.5(50–59), 5.5(60–69), 8.5(70–74)	*US $124,564*	
		· screen every 2 years 55–69	3.0 ng/ml	*US $120,952*	
		*· screen every 4 years 55–69*	*3.0 ng/ml*	*US $70,831*	

Italicised text indicates potentially cost-effective scenario. ^a^ Dominated; the strategy is more costly and less effective than the comparator (commonly, usual practice)

Most studies compared screening strategies to ‘no screen’ despite the relatively high prevalence in practice of background or opportunistic screening.

### Treatment types

All studies referred to a biopsy to confirm diagnosis, but only four studies detailed the type of biopsy - TRUS guided [22, 27, 29, 31]. Radical treatments, such as radiotherapy and prostatectomy with and without hormone therapy were considered, as well as conservative treatment. Four different terms were used to describe the strategy of delayed radical treatment, and few studies provided details on what it involved (Additional file 2). Five studies, three of which found strategies to be cost-effective, explicitly stated that men on conservative management eventually received radical treatment, but the approaches varied widely [23–25], with different percentages assumed, e.g. 30% of men receive treatment after 7 years in Heijnsdijk et al. [25] and 10% within 2 years in two studies [23, 24]. Whilst the other two studies, which found strategies to be cost-effective, based likelihood of progression to radical treatment on time spent in the disease state [29] or would-be clinical diagnosis in the absence of a screening programme [28].

In addition to comparing radical or conservative treatment following diagnosis, two studies also assessed cost-effectiveness of screening by risk-stratifying treatment [28, 29]. For example, men with low risk cancer receive conservative treatment until signs of progression, instead of immediate radical treatment. Keller et al. [29] found screening men aged 50–69 years old every 2 years and managing low risk men with active surveillance to be cost-effective ($45,882/QALY gained) and Roth et al. [28] reported a range of screening scenarios to be more cost-effective when selective treatment practices are employed when compared to opportunistic screening and no screening respectively (Table 2).

Keller et al. [29] did not report overdiagnosis, but noted that an active surveillance treatment strategy for low risk cancer could limit overtreatment.

### Model features

#### Model type

Cost-effectiveness models involved either a cohort-level (i.e. macrosimulation) or individual patient-level (i.e. microsimulation) modelling approach to estimate the expected costs and outcomes of screening.

Four Markov cohort models were identified [27, 29–31], where men with similar characteristics are grouped together and modelled as a cohort. Also, four individual patient level models [23, 25, 26, 28], where men are simulated individually to allow for variability across individuals were identified. Additionally, one ‘population based model’ [22], and one natural history model that was converted from a patient-level to a cohort model were also identified [24].

The model type did not depend on the screening strategy assessed, except the two studies that considered adaptive screening strategies were implemented using individual patient-level models, as these models more readily allow tracking of individual patients to, for example, recall only moderate to high risk men for a subsequent screen. Three of the four microsimulation models were epidemiological natural history models adapted to assess cost-effectiveness: The Microsimulation Screening Analysis (MISCAN) model [25] and the Fred Hutchinson Cancer Research Centre (FHCRC) model [26, 28]. These models estimate unobservable processes, such as the natural history of the disease, and are developed to evaluate the effectiveness of screening programmes or to provide simulated estimates of overdiagnosis.

#### Model pathways of prostate cancer

The structure of the model reflects the natural history of the condition and the impact of screening on disease progression. The natural history of prostate cancer, particularly progression from asymptomatic to clinically detectable disease, is not well understood and has been captured with differing degrees of detail across the models due to a lack of data (Table 3).

**Table 3:** Model characteristics

Study	Model-type	Natural history model	By (TNM) stage of cancer?	TNM staging used	Differentiation by Gleason grade?	Gleason grading used	Time horizon	Deterministic sensitivity analysis	Probabilistic sensitivity analysis
Chilcott et al. [23]	Individual patient simulation	No	Yes	Localised (T1–2), Locally advanced (T3–4) and metastatic	Yes	(G < 7, G = 7, G > 7)	lifetime	Yes	Yes
Heijnsdijk et al. [25]	Individual patient simulation	Yes -MISCAN	Yes	18 stages: each tumour stage (T1,2 etc) modelled individually	Yes	(G < 7, G = 7, G > 7)	lifetime	Yes	No
Hummel and Chilcott [24]	Individual patient simulation and cohort	No	Yes	Localised (T1–2), Locally advanced (T3–4) and metastatic	Yes	(G < 7, G = 7, G > 7)	lifetime	Yes	No
Keller et al. [29]	Cohort model	No	Yes	Low (≤T1a), intermediate (≤T2b), high risk (>T2b) and metastatic	Yes	G ≤ 6, G ≤ 7, G > 7	up to 70 years	Yes	Yes
Kobayashi et al. [27]	Markov cycle tree (cohort)	No	Yes	Localised (T1–2), Locally advanced (T3–4) and metastatic	No	–	up to 80 years old	Yes	No
Martin et al. [30]	Cohort model	No	No	None	No	–	50 years (lifetime)	Yes	No
Pataky et al. [26]	Individual patient simulation	Yes -adapted FHCRC	Yes	Locoregional (≤T2a vs > T2) distant disease	Assumed Yes^a^	Not reported	assumed lifetime	Yes	No
Roth et al. [28]	Individual patient simulation	Yes -adapted FHCRC	Yes	Locoregional (≤T2a vs > T2) distant disease	Yes^a^	Indirectly (8–10/ 2–7)	lifetime	Yes	Yes
Wolstenholme et al. [31]	Cohort model	No	Yes	Localised (T1–2), Locally advanced (T3–4) and metastatic	No	–	lifetime(100 years old)	Yes	Yes
Shteynshlyuger & Andriole, [22]	Population based model	–	Yes	No details provided	–	–	lifetime	Yes	No

^a^ Based on values derived from the natural history, but Gleason is not explicitly modelled in the model-based economic evaluation

All but one cohort level model followed the established TNM classification, but only one of these four studies incorporated the likelihood of disease progression through Gleason grade. Martin et al. [30] reported 4 yearly screening for high risk men to be cost-effective, but the model pathway did not consider stages or grades of cancer, only presence or absence of cancer. The cohort model by Keller et al. [29], which found screening followed by selective treatment to be cost-effective, was the only study to model prostate cancer according to the D’amico classification of low risk (G ≤ 6, PSA ≤ 10,≤T1a), intermediate (G ≤ 7, PSA ≤ 20, ≤T2b), high (G > 7,PSA > 20,>T2b), and advanced cancer.

All individual patient-level models followed the TNM classification and incorporated Gleason grade. Similar to two of the cohort models, two patient-level models combined stages of prostate cancer to represent localised (T1-T2), locally advanced (T3–4) and metastatic cancer [23, 24, 27, 31] and each subdivided Gleason grade into three categories (G < 7, G = 7 and G > 7). Another individual level model, which found single screens and repeat screens to be cost-effective, differentiated the TNM staging classification by allowing up to 18 stages (9 pre-clinical and 9 clinical states), where each tumour stage (T1, 2, 3,4) was modelled individually [25], but the rationale for such delineation was not provided. This was also the only study to allow for disease progression by Gleason grade within a stage [25]; however, the evidence or reasoning for this approach is unclear.

Two further individual-level models refer to loco-regional (partitioned by ≤T2a vs > T2) and distant disease states [26, 28]. The natural history model used to inform these cost-effectiveness models by Pataky et al. [26] and Roth et al. [28] link cancer progression to PSA levels and allow post-onset PSA values to differ depending on Gleason score (8–10 or 2–7). One of these individual models found repeat screening every 4 years and selective treatment following screening to be potentially cost-effective.

#### Assessing uncertainty in cost-effectiveness results

All studies conducted a deterministic sensitivity analysis (where one model input is changed and the others remain the same) to assess the robustness of the cost-effectiveness results to changes to model inputs [22–31]. Best case and worst case scenario analyses were conducted where: for example, the highest and lowest quality of life values were used for the health states [25, 26]. In their sensitivity analyses, all studies that showed a screening strategy to be potentially cost-effective found that the results were sensitive to the quality of life values used [25, 28–30].

Only two of the four cohort-level models and two individual patient-level simulation models conducted a more comprehensive assessment of uncertainty, a probabilistic sensitivity analysis [23, 28, 29, 31]. In a probabilistic sensitivity analysis, model inputs (e.g. likelihood of disease progression, screen detection, cost and quality of life values) are changed simultaneously according to an appropriate distribution of plausible values. Both Roth et al. [28] and Keller et al. [29], who found strategies to be cost-effective conducted a probabilistic sensitivity analysis, which showed that the strategies had a probability of approximately 50% or less of being cost-effective at decision-maker willingness to pay thresholds of $50,000/ QALY gained (US dollars and AUS dollars respectively). Roth et al. [28], also showed that the probability of cost-effective increases marginally if the decision-maker threshold is raised to $150,000/ QALY gained.

### Model inputs

#### Quality of life

The estimated impact on health-related quality of life for the same health state (e.g. advanced cancer) varied across the studies (Tables 4 and 5). Two of the ten studies did not incorporate quality of life [22, 31].

**Table 4:** Characteristics of quality of life values used

Study	Study setting	Perspective	Assessment of QoL	Population	Country
Chilcott et al. [23]	UK	NHS	HUI/EQ-5D	General population	UK/other
Heijnsdijk et al. [25]	NR	Healthcare based on included costs	SG/EQ-5D/TTO/VAS	Patients/experts/ general population	Netherlands, US, Canada
Hummel and Chilcott [24]	UK	NHS	HUI/EQ-5D	General population	UK/other
Keller et al. [29]	Australia	Healthcare	SF-12/ SF-36/ other^a, b^	General population	Australia/ Finland
Kobayashi et al. [27]	NR	Societal	TTO	Physicians/ patients	Unclear
Martin et al. [30]	Australia	Healthcare	SF-12/ SG	Patient/ General population	US (adjusted)/ Australia
Pataky et al. [26]	Canada	Healthcare based on included costs	SG	2 different patient groups	Canada
Roth et al. [28]	US	US payer perspective	SG	Patient	US

*HUI* Health utility index, *QoL* quality of life, *SG* standard gamble, *TTO* Time trade off. ^a^The exact source of the value for advanced disease is unclear, it is likely to reflect a synthesis of EQ-5D and 15D.^b^ assumed SF measures converted to SF-6D

**Table 5:** Quality of life values assigned to health states

Study	Starting state	Diagnosis		Treatment		Other	Advanced	End of life	Adverse effects
		Biopsy	Cancer	Short- term	Long-term				
Chilcott, Hummel [23]	Baseline = age dependent	–	–				0.635		Bowel function = 0.89 Urinary function = 0.94 Sexual dysfunction =0.9
Heijnsdijk et al. [25]	Screening = 0.99	0.9	0.8	Radiation = 0.73	Radiation = 0.78	Post-recovery = 0.95	0.6	0.4	Short-term & long-term effect
				Prostatectomy = 0.67	Prostatectomy = 0.77				
				Active surveillance = 0.97	Active surveillance = 0.97				
Hummel and Chilcott [24]	Baseline = age dependent						0.635		Bowel function = 0.89 Urinary function = 0.94 Sexual dysfunction =0.9
Keller et al. [29]	age dependent/ screening = 1.0			0.95^b^	0.95^b^		0.9 to >0.6^b^		See treatment: Persistent effects, 3 years post-diagnosis
Kobayashi et al. [27]						Curable = 0.9 Recurrent = 0.7	0.5		See curable: impotence & incontinence
Martin et al. [30]			0.95^a^					0.5	See cancer
Pataky et al. [26]	Healthy screening population = 1.0			0.88	0.9	Symptomatic =0.9	0.85	0.5	Short-term & long-term effect
Roth et al. [28]	Healthy screening population = 1.0			0.75	0.92	Symptomatic =0.89	0.75	0.33	Short-term & long-term effect
				Surveillance = 0.92	Surveillance = 0.92				

^a^ Diagnosed and treated. ^b^multipliers of age dependent baseline value

Evidence on quality of life impact was taken only from secondary sources. The methods used varied across the studies. Only three studies exclusively used quality of life values from the same country context [26, 28, 30]; the remaining studies combined values from different countries and settings. None of the studies followed recommended methods to estimate quality of life: values that are derived using the same method in the same population group across all the model health states [10], and quality of life scores that are based on values derived by the general population [10–16].

Of the four studies that found screening to be cost-effective, three used *some* quality of life values that were based on recommended measures, EQ-5D [25] and SF-6D [29, 30]. However, scores from different measures were combined and two studies respectively used different country contexts [25, 29] values or used both patient and general population values [25, 30]. Although recommended measures were not used and values were not based on the general population, Roth et al. [28] was the only study to use the same method across all health states and values from the same population in the same country.

#### Adverse effects

Both Chilcott et al. [23] and Pataky et al. [26] highlighted that overdiagnosis and overtreatment are captured by quality of life, through adverse effects [23] and the additional men overtreated and treated earlier [23, 26]. Although neither Wolstenholme et al. [31] or Shteynshlyuger & Andriole [22] captured quality of life, they discussed that any benefits of life years gained reported were likely to be outweighed or overestimated due to the quality of life impacts related to adverse treatment effects.

All cost-utility analyses applied quality of life values that relate directly or indirectly to adverse effects. The primary adverse effects were considered: urinary, sexual and bowel problems following treatment. Two studies explicitly applied a quality of life decrement for these adverse effects [23, 24], whilst the remaining studies applied quality of life values for a post-treatment effect, which includes patients who did and did not experience adverse effects.

Three studies, two of which showed strategies to be cost-effective, used different quality of life values for ‘short-term’ and ‘long-term’ treatment effects, which implicitly allows for the adverse effects associated with treatment [25, 26, 28]. Though there appears to be no consensus on the length of time that constitutes a short or long-term effect. Two studies, one which showed strategies to be cost-effective, referred to short-term treatment as the first 2 months post-treatment [25, 26] and the third study, which showed strategies to be cost-effective, referred to short term as up to one-year post-treatment [28]. Of the remaining two studies that showed strategies to be potentially cost-effective, Martin et al. [30], applied a quality of life score to ‘prostate cancer’ (diagnosis and treatment), which was a decrement averaged over the survival period, obtained from patients who experienced urinary, bowel and sexual problems, and Keller et al. [29] considered only persistent adverse effects 3 years post-diagnosis.

#### Resource use

Seven studies (including three of the four studies which showed strategies to be cost-effective) took a healthcare perspective for the analysis [23–26, 29–31], including the cost of procedures, equipment and resource use (staff time) associated with the screening test, biopsy, treatments, GP visits, hospital admissions and procedures, as well as terminal care costs. Two studies took a societal perspective, which includes direct healthcare costs as well costs to patients, carers, and other sectors (such as lost productivity), but costs were not explicitly reported [22, 27], making it difficult to assess the appropriateness of the included costs. The final US study, which showed strategies to be cost-effective, took a US payer perspective [28], where healthcare perspective costs were included, but the inclusion of out of pocket costs to patients was unclear. Costs associated with treatment complications were explicitly excluded in two studies [23, 24], explicitly included in two studies (one of which showed strategies to be cost-effective) [28, 31], with the Keller et al. study including only immediate complications [29], it is unclear whether they were included in the remaining studies.

## Discussion

Several model-based economic evaluations assessing many different PSA screening strategies were identified. Strategies ranging from different PSA thresholds, screening intervals, and age cohorts of men were assessed. Four of the ten studies identified screening strategies that could be potentially cost-effective including; delayed radical treatment for low risk cancer, single screen at 55 years old (3.0 ng/ml), annual or two-yearly screens at 55 years old (3.0 ng/ml) with stopping ages between 59 and 63 years old, screening very high-risk men every 4 years (4.0 ng/ml), and screening men aged 55–69 years old every 4 years (10 ng/ml). However, these studies found the results to be sensitive to the quality of life values used and revealed a lack of good quality data for the country context to inform the cost-effectiveness analysis.

### Similarities, deviations and key issues for future models

Most studies considered TNM and approximately half considered Gleason grade to model prostate cancer progression, but the degree to which TNM and Gleason grade was incorporated varied across the studies. This may reflect the lack of agreement or changing trends overtime on how best to represent prostate cancer progression. Extensive clinical input and exploration of alternative model structures in sensitivity analyses to categorise prostate cancer could alleviate these issues, but both disease stage and grade should be incorporated, as these characteristics inform treatment decisions [32].

There was no consensus on the optimal model type for modelling prostate cancer screening. Adaptive screening, where subsequent screens are based on risk stratification, was modelled using individual-patient level models. This model-type readily accounts for the complexity of the disease natural history and the requirements to model adaptive screening strategies, but additional data and model assumptions are required to populate these models. The increased flexibility in analysis should not result in more trust being placed in the model than is warranted, where limited country-specific empirical evidence is available, as these individual-level models are unlikely to increase the accuracy of cost-effectiveness results. When adaptive screening is not considered, cohort-level modelling may be more suitable to evaluate the cost-effectiveness of prostate cancer screening. Further, when making recommendations to decision-makers, it is crucial to estimate uncertainty associated with the model inputs used, regardless of model type used. Fewer than half the studies conducted the more comprehensive assessment of uncertainty to assess the robustness of the cost-effectiveness result to changes in model inputs.

Most studies incorporated quality of life to account for the impact on morbidity of screening and treatment, which is recognised as an appropriate method for capturing the impact of overdiagnosis and overtreatment. However, recommended methods for capturing quality of life were rarely followed and the combination of different values derived from different measures from different populations impacts on the meaningfulness of the results, as the measures and values from different populations are not comparable.

Several studies, three of which showed strategies to be cost-effective, may be at risk of overestimating the benefit of screening by not considering opportunistic or background screening, which varies considerably across countries. To ensure that the cost-effectiveness results are appropriately estimated, the comparator of no systematic screen should include opportunistic screening. Finally, given the high rates of overdiagnosis and overtreatment in prostate cancer, the cost and quality of life associated with screening, treatments and adverse events should be incorporated in a cost-effectiveness analysis to capture the impact of overdiagnosis and overtreatment.

### Strengths and limitations

This systematic review provides updated evidence on the modelling methods used to date to assess the cost-effectiveness of prostate cancer screening. Three reviews in the area were identified [23, 33, 34], but two did not address the specific review question posed here (see Additional file 1) and the other is now 8 years old. A limitation of the current review is that a comparison between the modelling outputs was not possible because no two modelling methods, treatment options, or outcome measures were the same or assessed in the same country context.

## Conclusion

The answer to the question of whether screening for prostate cancer is cost-effective is unclear. Despite several model-based evaluations, robust evidence to inform cost-effectiveness is lacking. Current country-specific data are required, along with prospective quality of life data that are incorporated into clinically verified models using recommended methods. Any recommendations to decision-makers should be comprehensively tested for uncertainty in model inputs.

## Additional files

Additional file 1: Search Strategy Methods. Further details on eligibility criteria, search terms, study selection process, data extraction, quality assessment and data synthesis methods. (DOCX 26 kb)

Additional file 2: Term used for conservative management. A summary table of the terms and definitions of conservative management used (DOCX 12 kb)

## Abbreviations

FHCRC: Fred Hutchinson Cancer Research Centre
ICERs: Incremental cost-effectiveness ratios
MISCAN: The microsimulation screening analysis
NICE: National Institute of Health and Care Excellence
PSA: Prostate-specific antigen
QALYs: Quality-adjusted life years
