# Supplementary material for: Cost-effectiveness of prostate cancer screening: a systematic review of decision-analytical models
Source: BMC Cancer. 2018 Jan 18;18:84. doi: 10.1186/s12885-017-3974-1 (PMC5773135; doi:10.1186/s12885-017-3974-1)
Supplement: Supplementary file 1 — Search Strategy Methods. Further details on eligibility criteria, search terms, study selection process, data extraction, quality assessment and data synthesis methods. (DOCX 26 kb) [file 12885_2017_3974_MOESM1_ESM.docx]

**Additional File 1.**

**Search strategy methods**

Methods of analysis and inclusion criteria were specified in advance and documented in a protocol (URL: <http://hdl.handle.net/1983/ac8fab64-ff2a-46a4-a2a2-fca30aab61b3>). An initial scoping search was carried out in March 2016 to identify existing reviews and the most appropriate search terms. Three publically available reviews were identified [23, 33, 34]. However, these reviews did not provide up-to-date evidence or answer the specific review question here.

The guidelines by the Centre for Reviews and Dissemination and Cochrane collaboration for reviews were followed [18, 19]. The review was restricted to evidence from the last 10 years (January 2006) to reflect current practice both in screening for prostate cancer and economic evaluation modelling methods.

***Eligibility criteria***

Studies were included if they met the following criteria:

- Model-based economic evaluation of PSA screening strategies for prostate cancer
- Comparator of no screen or any screening interval
- Cost-effectiveness, cost-utility analysis, cost-consequence analysis, cost-benefit analysis
- Model-based economic evaluation using primary or secondary data
- Any PSA threshold for determining a positive result
- Any subsequent treatments following the PSA screen
- Any country and context
- An outcome of QALYs or life years gained/saved
- Natural history models of prostate cancer that did not include an economic evaluation, but were used to inform the model structure

***Search strategy***

*Data sources*

In April 2016, studies were identified by searching electronic databases (listed below), NICE guidelines, UK National Screening Committee guidance, reference lists from relevant studies and contacting experts. The search was limited to English language publications and studies published between January 2006 and April 2016. An update of the literature search was performed from April 2016-February 2017.

The search was applied to the NHS Economic Evaluation Database (EED) (2006-2014), Medline (2006-2016), EMBASE (2006-2016) and HTA databases (2006-2016). The NHS EED the database no longer runs searches of other databases to identify economic evaluations, the last search was at the end of 2014.

Reports from NICE and UK National Screening Committee were considered as they are important inputs to UK decision-making and can inform practice in other countries.

*Search terms*

Search terms included prostate cancer **and** screening **or** testing **and** economic evaluation **or** cost-effectiveness **or** natural history **or** screen models and their variants (see example below).

The search strategy consisted of MeSH terms and free text terms. Search terms for economic evaluation and prostate cancer screening were based on: previous reviews [23, 36]; key words used in the known model-based economic evaluations of prostate cancer; and those used by CRD to retrieve economic evaluations.

### Ovid - Medline

1. exp prostatic neoplasms/

2. (cancer adj3 (prostate or prostatic)).tw.

3. (carcinoma adj3 (prostate or prostatic)).tw.

4. (neoplas$ adj3 (prostate or prostatic)).tw.

5. (malignan$ adj3 (prostate or prostatic)).tw

6. (prostat$ adj3 (neoplasm$ or cancer or carcinoma or tumo?r$ or malignan$)).tw

7. 1 or 2 or 3 or 4 or 5 or 6

8. Prostate-Specific Antigen/

9 (prostate specific antigen or prostate-specific antigen or psa) tw

10. Mass screening/

11. (Screen$ or test$) tw

12. 8 or 9 or 10 or 11

13. exp “costs and cost analysis”/

14. (model adj3 (economic or cost)).tw.

15. (cost adj3 (effect$ or util$)).tw.

16. (economic adj3 (anal$ or eval$)).tw.

17. (natural history model) tw

18. (screen$ model$) tw

19. (disease progression model$) tw

20. 13 or14 or 15 or 16 or 17 or 18 or 19

21. 7 and 12 and 20

22. limit 12 to yr=”2006-Current”

*Study selection*

Eligibility assessment was performed independently by two reviewers in two stages:

Firstly, the titles and abstracts of the identified studies were assessed against the eligibility criteria to identify potentially relevant papers (SS). 10% of the titles and abstracts were reviewed by a second reviewer (SM). Where it was unclear if a study should be included it was carried forward to the next stage and if an abstract was not provided the full text of the paper was retrieved.

Secondly, the full text of potentially relevant papers was screened. Relevant studies were carried forward for data extraction. All of the full text papers were reviewed by a second reviewer (SM). Studies were categorised according to (a) model-based economic evaluation for screening in prostate cancer, (b) natural history models that inform the model structure. Disagreements about study eligibility were discussed.

*Data* *extraction*

Data extraction forms were developed and pilot-tested on a random sample (5%) of included studies, and refined accordingly. Data extraction was performed independently by two reviewers (SS, SM). Disagreement was resolved by discussion between the two review authors.

Information was extracted from each included study on:

1. The policy question and context (including the comparators and country)
2. Characteristics of the screening strategy (including frequency of screening, starting age and PSA threshold for a positive result)
3. Type of treatments and biopsies
4. Type of outcome measure (including cost per QALY gained and life-year gained)
5. Cost-effectiveness result
6. Characteristics of the model (including model type, structure, handling of disease natural history)
7. Sensitivity analysis (including the extent to which uncertainty in the cost-effectiveness result had been quantified.
8. Evidence base for quality of life, resource use and adverse effects
9. Reporting of overdiagnosis and overtreatment
10. Other key areas included quantification of overdiagnosis and overtreatment, the clinical basis for the model (stage or grade progression), and the method for incorporating quality of life and resource use.

*Quality assessment*

As the purpose of the review was to report the methodological approaches used in model-based economic evaluations, a formal quality checklist was not used to exclude studies from the review. However, relevant sections of an existing economic evaluation checklist along with recommendations from NICE guidance were used to report the studies [10, 21]. In addition, key clinical issues that are known to be a concern in prostate cancer were included, such as reporting and capturing of overdiagnosis. The methodological components that were assessed include appropriateness of: (1) screening strategies and treatments considered, (2) reporting of overdiagnosis and overtreatment (3) appropriateness of model inputs, (4) consistency of model structure with disease pathway theory, (5) model type and justification, (6) time horizon of analysis, (7) cycle length and justification, (8) model inputs and data modelling (including baseline data, cost and quality of life), (9) assessment of uncertainty.

*Data synthesis*

Due to the nature of the review, a narrative synthesis of data was undertaken and not a meta-analysis. A discussion reflecting the modelling methods used and the impact on the cost-effectiveness is provided. As the purpose of the review is to identify key issues in modelling screening for prostate cancer and the types of modelling methods used, the quality of the studies is not assessed in relation to deciding whether they are included in the review. Rather, analysis of quality issues will form part of the synthesis.
