# Supplementary material for: Cost-effectiveness of prostate cancer screening: a systematic review of decision-analytical models
Source: BMC Cancer. 2018 Jan 18;18:84. doi: 10.1186/s12885-017-3974-1 (PMC5773135; doi:10.1186/s12885-017-3974-1)
Supplement: Supplementary file 2 — Term used for conservative management. A summary table of the terms and definitions of conservative management used (DOCX 12 kb) [file 12885_2017_3974_MOESM2_ESM.docx]

**Additional File 2.**

**Table S1**. Term used for conservative management

| **Study** | **Term used** | **Meaning** |
| --- | --- | --- |
| Chilcott et al. (2010) | Active monitoring (<70 years old) | Monitor PSA 4 times a year/ biopsy every 2 years |
|  | watchful waiting (>70 years) | GP twice a year and PSA test visit |
| Heijnsdijk et al. (2015) | Active surveillance | A series of multiple tests (PSA, DRE and biopsies) |
| Hummel and Chilcott (2013) | Active monitoring (<70 years old) | Monitor PSA 4 times a year/ biopsy every 2 years |
|  | watchful waiting (>70 years) | GP twice a year and PSA test visit |
| Keller et al. (2017) | Active surveillance (<76 years) | Surveillance with curative intent |
|  | watchful waiting (≥76 years old) | Surveillance with palliative intent, 2 specialist consultations and 2 PSA tests |
| Kobayashi et al. (2007) | No details | - |
| Martin et al. (2013) | No details | - |
| Pataky et al. (2014) | Conservative management | No details |
| Roth et al. (2016) | Conservative management | Annual office visits & PSA test/ biennial biopsy |
| Wolstenholme et al. (2011) | Active surveillance | Outpatient consultation, PSA test and biopsy 4 times a year in year 1 and 2, and then 2 from year 3 onward |
| (Shteynshlyuger & Andriole, 2011) | No details | - |
